# Association of antenatal dexamethasone administration timing with outcomes in preterm infants in a low- and middle-income country

**DOI:** 10.3389/fmed.2025.1712437

**Published:** 2025-11-27

**Authors:** Vijay Kalrao, Mamta Benda

**Affiliations:** Department of Pediatrics, Bharati Vidyapeeth Medical College Hospital, Pune, Maharashtra, India

**Keywords:** preterm birth, neonatal outcomes, neonatal mortality, necrotizing enterocolitis, antenatal corticosteroid timing, dexamethasone dose-to-delivery-interval, low- and middle-income countries, propensity score

## Abstract

**Background:**

Timely administration of antenatal corticosteroids improves preterm outcomes; however, in low- and middle-income countries (LMICs), multiple barriers often delay treatment. Whether exposure within 24 h before delivery confers benefits remains uncertain. We aimed to evaluate the association between antenatal dexamethasone-to-delivery intervals and preterm outcomes in an LMIC setting.

**Methods:**

This single-center prospective cohort study was conducted at a tertiary neonatal intensive care unit in India from June 2023 to December 2024. The participants were preterm infants born at 24 0/7–33 6/7 week gestation. Exclusion criteria were major congenital anomalies, multiple courses of dexamethasone, administration-to-birth interval > 7 d, and interfacility transfer. The exposure variable was maternal first dexamethasone dose-to-delivery interval, categorized as ≤ 24 h (*n* = 167), > 24 h–7 d (*n* = 64), or no exposure (*n* = 101). The primary outcome was in-hospital mortality. Secondary outcomes included severe necrotizing enterocolitis (NEC), intraventricular hemorrhage, respiratory distress syndrome, and a composite of death or severe morbidity. Propensity scores from 10 covariates generated inverse-probability-of-treatment-weights (IPTW). Adjusted risk ratios (aRRs) and 95% confidence intervals (CIs) were estimated using a doubly-robust, trimmed IPTW-modified Poisson model. Sensitivity analyses used doubly-robust overlap weighting and an entropy-balanced treatment-only model.

**Results:**

Of the 371 infants born during the study period, 332 met eligibility criteria (mean gestational age, 30.6 ± 2.2 week; 60.5% male). Overall mortality was 7.8% (26/332): 11.9% (12/101) without dexamethasone, 6.6% (11/167) with ≤ 24 h exposure, and 4.7% (3/64) with > 24 h–7 d exposure. Mortality did not differ by dexamethasone timing intervalgroup: ≤ 24 h (aRR, 0.58; 95%CI 0.26–1.27) and > 24 h–7 d (aRR, 0.33; 95%CI, 0.10–1.12) vs. no dexamethasone. Wide CIs were observed, reflecting imprecision due to lower-than-expected mortality in the unexposed group. Severe NEC was lower with ≤ 24 h exposure (1.8% [3/167] vs. 8.9% [9/101]; aRR, 0.15; 95%CI 0.04–0.54), persisting in sensitivity analyses (aRR, 0.11; 95%CI 0.03–0.38) and after multiple-comparison adjustment. No other secondary outcomes differed significantly.

**Conclusion:**

Dexamethasone administration within 24 h before delivery did not reduce mortality but significantly decreased the risk of severe NEC. Imprecise estimates due to lower-than-expected mortality, reduced statistical power, and potential residual confounding limit definitive conclusions.

## 1 Introduction

Preterm birth affects over 15 million infants annually worldwide, with low- and middle-income countries (LMICs) bearing a disproportionate burden and higher mortalities (1). India has the highest number of preterm births globally, with premature birth causing 46.1% of neonatal deaths (1, 2). Thus, evidence-based interventions tailored to these settings are required.

Antenatal corticosteroids (ANCS) have been integral to preterm birth management since 1972, reducing respiratory distress syndrome (RDS), intraventricular hemorrhage (IVH), necrotizing enterocolitis (NEC), and mortality (3–5). The World Health Organization (WHO) and American College of Obstetricians and Gynecologists guidelines recommend ANCS administration in women at risk of preterm delivery between 24 and 34 week of gestation (6, 7). The optimal interval between ANCS administration and delivery ranges from 24 h to 7 day (8–10). However, meta-analyses show heterogeneity in the reported optimal ANCS administration timing, underscoring the uncertainty regarding maximal benefit intervals (11).

Adherence to the optimal interval in LMICs is challenging because of delayed presentation, rapid labor progression, transportation barriers, and inconsistent availability of medications (12, 13). Evidence from high-resource settings suggests the advantages of shorter exposure intervals (≤ 24 h) to betamethasone (14–16). A secondary analysis of the WHO ACTION-I trial in LMICs showed a non-significant reduction in mortality with dexamethasone administered within 12–36 h, a cost-effective option in limited-resource settings (17). These benefits have been attributed to the rapid non-genomic effects of glucocorticoids (18).

Most studies on ANCS administration timing have been conducted in high-resource settings (15, 16), limiting the applicability of their findings to LMICs, which differ substantially from high-resource settings in terms of mortality rates, healthcare infrastructure, and co-interventions (11, 13). In India, implementation of the ANCS policy has faced systemic barriers despite national protocols being introduced in 2014 (13, 19).

Relevant studies often lack precision in assessing rare but significant outcomes owing to small sample sizes, and their observational designs are susceptible to confounding. Studies from LMICs and high-resource settings have not assessed the effects of the ANCS-to-delivery interval in broader preterm populations, often excluding women with infections (17) and focusing on narrow gestational or timing windows (15). In LMICs, where most preterm births occur late in labor and receive dexamethasone within 24 h of delivery owing to barriers to timely care (13), efficacy data for such short exposures remain limited.

This observational study, conducted in a tertiary neonatal intensive care unit (NICU) in India, investigated the association between antenatal dexamethasone administration-to-delivery interval and neonatal outcomes in preterm deliveries at 24–34 week of gestation, including in women with clinical infection, under real-world practice conditions. We hypothesized that a ≤ 24-h interval would be associated with reduced mortality and morbidity compared with no steroid exposure, providing LMIC-specific evidence for short-exposure efficacy.

## 2 Materials and methods

### 2.1 Study setting and design

This prospective cohort study was conducted between June 2023 and December 2024 in a 40-bed level-III NICU within a tertiary medical college hospital in India. This study was approved by the Institutional Ethics Committee (Approval No. BVDUMC/IEC/2023/44) and adhered to the STrengthening the Reporting of OBservational studies in Epidemiology guidelines. Written informed consent was obtained from the parents.

### 2.2 Participants

Of 371 screened neonates, 332 infants born between 24 0/7 and 33 6/7 week gestation and admitted within 24 h of birth were enrolled; 39 were excluded due to major congenital anomalies (*n* = 8), receipt of > 1 ANCS course (*n* = 9), administration-to-delivery interval > 7 d (*n* = 12), or interfacility transfer before outcome assessment (*n* = 10).

### 2.3 Exposure

ANCS administration followed national guidelines recommending intramuscular dexamethasone (6 mg every 12 h × four doses) (19). Shared obstetric–neonatal electronic records enabled deterministic mother–infant linkages. ANCS exposure was defined as the administration of maternal dexamethasone before delivery. The interval from the first-dose-to-delivery was determined through maternal interviews, medical records, and labor logs, and verified by two independent clinicians. Intervals were categorized as ≤ 24 h, > 24 h–7 day, or no exposure. Exposure was defined as the interval from the first ANCS dose to delivery rather than from the last dose, to reflect the initiation of the steroid effect, minimize post-exposure bias arising from last-dose scheduling near imminent delivery, and align with clinical timestamps that are reliably recorded in practice. Dexamethasone administration ≤ 24 h before delivery likely reflects a more urgent clinical scenario, potentially introducing residual confounding despite statistical adjustment.

### 2.4 Outcomes

The primary outcome was the in-hospital mortality rate. Secondary outcomes included severe morbidities defined by standardized criteria: severe RDS (radiographic and clinical grade ≥ 3), severe IVH (Papile grade ≥ 3), severe NEC (Bell stage ≥ 2A), and a composite outcome of death or any severe morbidity. Low event rates produce wide confidence intervals (CIs) and limited precision, constraining reliability, particularly for rare but critical outcomes.

### 2.5 Covariates

Ten baseline variables were selected *a priori* as potential confounders based on biological plausibility or observed baseline imbalances (15–17): gestational age (GA) at birth, birth weight (continuous), infant sex, small-for-gestational-age status, plurality (twin gestation), pre-eclampsia, preterm premature rupture of membranes, clinical chorioamnionitis, nulliparity, and inducedpreterm delivery. These upstream clinical indications (induced preterm delivery, preterm premature rupture of membranes, and pre-eclampsia) precipitate delivery mode and urgency, thereby addressing confounding by indication related to cesarean or vaginal delivery.

### 2.6 Data collection

Data were prospectively collected by trained research assistants using a piloted case report form. Maternal data included demographics, obstetric history, antenatal care, comorbidities, pregnancy complications (pre-eclampsia, preterm premature rupture of membranes, chorioamnionitis, etc.), delivery mode, and ANCS use. Neonatal data were recorded daily from birth until discharge/death, including GA (per early ultrasonography or last menstrual period, corroborated by the Ballard score), birth weight, sex, Apgar scores, resuscitation, NICU therapies, and outcomes. A senior neonatologist independently reviewed 10% of the records to ensure accuracy. All 332 included infants had complete data for primary covariates and outcomes, eliminating the need for imputation or complete-case sensitivity analyses.

### 2.7 Statistical analysis

*A priori* calculations indicated that 276 infants (184 exposed, 92 unexposed; 2:1 allocation) would provide 80% power (two-sided α = 0.05) to detect a mortality reduction from 20 to 8% with dexamethasone (14). The observed mortality rate in the unexposed group was lower (11.9%), yielding wide CIs and imprecise risk estimates.

Categorical variables were compared using χ^2^ or Fisher’s exact test, and continuous variables were compared using analysis of variance or the Kruskal–Wallis test. The results are reported as mean (standard deviation), median (interquartile range), or proportion (*n* [%]).

Propensity score-based weighting addresses confounding factors by indication. The probability of assignment to one of three exposure categories (no exposure, first dexamethasone dose-to-delivery interval ≤ 24 h, or > 24 h–7 day) was estimated using a multinomial logistic regression with heteroskedasticity-consistent (HC1) robust variance, including 10 pre-specified baseline covariates. Two weighting schemes were derived from the predicted class probabilities (p̂) obtained via the softmax of the fitted coefficients.

The stabilized inverse-probability-of-treatment weights (IPTW) was calculated as the marginal prevalence of the observed exposure divided by p̂, then (i) trimmed at the 1st and 99th percentiles, (ii) rescaled to a mean of 1, and (iii) capped at 20. This targets the average treatment effect in the full cohort while controlling for extreme weights. After trimming, the groups categorized based on the dexamethasone-to-delivery interval showed a strong distributional overlap.

Overlap weights (OW) were defined as 1-p̂g for the infant’s exposure category (g), emphasizing that infants with covariate profiles that made all timing categories similarly likely and targeted the clinical-equipoise sub-population (20).

Absolute standardized mean differences (SMDs) were used to assess covariate balance (excellent, < 0.10; acceptable, < 0.25) (20, 21). SMDs were calculated before weighting, after trimmed/stabilized IPTW, and after OW. The covariate balance before and after weighting, full pair-wise SMDs, and predictors of antenatal corticosteroid–to–delivery time are shown in Table 1, Supplementary Table 1, and Table 2; maximum covariate-level SMDs after IPTW and OW are summarized in Supplementary Tables 2, 3.

**TABLE 1:** Baseline characteristics and covariate balance before and after weighting.

Covariate characteristic	No exposure	ANCS-to-delivery interval ≤ 24 h	ANCS-to-delivery interval > 24 h–7 d	*P* value	Abs SMD unweighted	Abs SMD post-IPTW	Abs SMD post-IPTW trim	Abs SMD post OW
Gestational age mean (SD), week	30.69 (2.43)	30.68 (2.13)	30.25 (2.14)	0.378	0.19	0.04	0.04	0.14
Birth weight, mean (SD), g	1440.75 (428.97)	1429.46 (425.33)	1405.47 (456.02)	0.877	0.08	0.04	0.03	0.07
Female sex	44 (43.6%)	66 (39.5%)	21 (32.8%)	0.387	0.22	0.02	0.01	0.16
SGA	9 (8.9%)	28 (16.8%)	8 (12.5%)	0.184	0.24	0.04	0.03	0.08
Twin pregnancy	18 (17.8%)	49 (29.3%)	16 (25.0%)	0.108	0.27	0.1	0.08	0.09
Pre-eclampsia	22 (21.8%)	57 (34.1%)	18 (28.1%)	0.096	0.28	0.1	0.08	0.07
PPROM	20 (19.8%)	41 (24.6%)	17 (26.6%)	0.547	0.16	0.02	0.01	0.09
Clinical chorioamnionitis	1 (1.0%)	5 (3.0%)	3 (4.7%)	0.344	0.22	0.09	0.1	0.17
Nulliparity	51 (50.5%)	88 (52.7%)	30 (46.9%)	0.727	0.07	0.05	0.04	0.05
Induced preterm delivery	19 (18.8%)	39 (23.4%)	16 (25.0%)	0.581	0.15	0.07	0.05	0.1

Abs SMD, absolute standardized mean difference; IPTW, inverse-probability-of-treatment weighting; OW, overlap weighting; PPROM, preterm premature rupture of membranes; SGA, small for gestational age. Continuous variables are expressed as mean (standard deviation, SD), and categorical variables are expressed as numbers (percentages). Abs SMD (Unweighted, IPTW, IPTW trim, OW) represents the larger of the two pair-wise contrasts (No ANCS vs. ≤ 24 h and No ANCS vs. > 24 h–7 d). Balance was considered excellent at | SMD| < 0.10 and acceptable at | SMD| < 0.25. Weighted means and proportions were calculated using stabilized IPTW (trimmed at the 1st–99th percentiles for the IPTW trim column). *P*-values were derived from one-way analysis of variance (continuous variables) or χ^2^ test/Fisher’s exact test (categorical variables).

**TABLE 2:** Predictors of antenatal corticosteroid–to–delivery time in the multinomial propensity score model.

Covariate	ANCS-to-delivery interval ≤ 24 h OR (95% CI)	ANCS-to-delivery interval > 24 h–7 d OR (95% CI)
Gestational age, mean SD, week	0.92 (0.76–1.11)	0.82 (0.66–1.03)
Birth weight mean SD, g	1.00 (1.00–1.00)	1.00 (1.00–1.00)
Female sex	0.82 (0.48–1.38)	0.61 (0.32–1.20)
SGA	2.01 (0.83–4.86)	1.75 (0.54–5.66)
Twin pregnancy	1.95 (0.99–3.84)	1.58 (0.69–3.65)
Pre-eclampsia	2.23 (1.04–4.76)	1.50 (0.62–3.63)
PPROM	1.63 (0.83–3.22)	1.54 (0.66–3.59)
Clinical chorioamnionitis	2.55 (0.30–22.05)	4.41 (0.45–42.97)
Nulliparity	1.14 (0.68–1.90)	0.89 (0.47–1.71)
Induced preterm delivery	1.10 (0.51–2.35)	1.59 (0.69–3.69)

PPROM, preterm premature rupture of membranes; SGA, small for gestational age; OR, odds ratio; ANCS, antenatal corticosteroids. Odds ratios > 1 indicate a higher likelihood of receiving ANCS in the specified timing category (≤ 24 h or > 24 h–7 d) vs. no ANCS, and ORs < 1 indicate a lower likelihood. Estimates come from a multinomial logistic regression (statsmodels MNLogit) with HC1 robust variance in the complete-case sample. Class probabilities from this model were used to construct the IPTW (stabilized, 1st–99th percentile trimmed, mean-rescaled, cap 20) and overlap weights (1 minus p observed). 95% CIs were computed as exp(beta ± 1.96 × SE). The strongest predictors of treatment timing before weighing were small for gestational age (SGA), pre-eclampsia, and clinical chorioamnionitis.

Weight diagnostics (Supplementary Table 4) confirmed stability: IPTW mean = 1.00, maximum = 2.19, effective sample size = approximately 306 (vs. 332 unweighted); OW mean = 1.00, maximum = 0.90, effective sample size = approximately 311. Trimmed/stabilized IPTW achieved the tightest balance (all pair-wise SMDs < 0.10) while preserving the full-cohort estimand and providing double robustness when combined with covariate adjustment; therefore, it served as the primary analytic weight.

This design addressed several anticipated biases: confounding by indication through propensity score weighting to achieve covariate balance (SMDs < 0.10), immortal time bias by defining exposure at actual delivery rather than intended intervals, and selection bias through high inclusion rates (89.4% of screened infants).

Primary outcomes were analyzed using doubly robust, trimmed, and stabilized IPTW-modified Poisson regression with HC3 (heteroskedasticity-consistent with leverage adjustment) robust variance estimates (Table 3). Weighted Poisson regression models were used to estimate treatment effects, treating all observations as independent despite 83 infants (25.0%) being from twin pregnancies, owing to the absence of maternal identifiers. The models included indicators for ≤ 24 h vs. no exposure and > 24 h–7 day vs. no exposure, along with the 10 covariates, including GA at birth, to adjust for heterogeneity across the 24–34 week range. This approach yielded unbiased adjusted risk ratios (aRRs) if either the propensity or outcome model was correctly specified (22). The same modeling strategy was applied to the secondary outcomes. For severe NEC, event counts in the ≤ 24 h group were sparse (three events in total: one at ≤ 12 h and two at 13–24 h), precluding subdivision by narrower timing bands. The resulting estimates, therefore, reflect the effect of early ANCS initiation under real-world conditions rather than completed-course effects.

**TABLE 3:** Association of first antenatal corticosteroid dose-to-delivery interval with neonatal outcomes after doubly robust inverse-probability weighting.

Outcome	No exposure Events n/total (%)	ANCS-to-delivery interval ≤ 24 h Events n/total (%)	ANCS-to-delivery interval > 24 h–7 d Events n/total (%)	Adjusted RR^a^ ANCS-to-delivery interval ≤ 24 h	Adjusted RR ANCS-to-delivery interval > 24 h–7 d	ARD^c^ ANCS-to-delivery interval ≤ 24 h (95% CI)	ARD^c^ ANCS-to-delivery interval > 24 h–7 d (95% CI)	NNT^d^ ANCS-to-delivery interval ≤ 24 h; > 24 h–7 d
Mortality	12/101 (11.9%)	11/167 (6.6%)	3/64 (4.7%)	0.58 (0.26–1.27)	0.33 (0.10–1.12)	−0.053 (−0.126 to 0.021)	−0.072 (−0.154 to 0.010)	19; 14
Severe RDS	34/101 (33.7%)	55/167 (32.9%)	23/64 (35.9%)	0.94 (0.68–1.29)	0.93 (0.62–1.38)	−0.007 (−0.124 to 0.109)	0.023 (−0.127 to 0.172)	NA; NA
Severe NEC	9/101 (8.9%)	3/167 (1.8%)	4/64 (6.2%)	0.15 (0.04–0.54)^b^	0.52 (0.16–1.67)	−0.071 (−0.130 to −0.012)	−0.027 (−0.108 to 0.055)	14; 37
Severe IVH	4/101 (4.0%)	4/167 (2.4%)	2/64 (3.1%)	0.84 (0.15–4.56)	0.74 (0.16–3.40)	−0.016 (−0.060 to 0.029)	−0.008 (−0.065 to 0.049)	NA; NA
Composite outcome	43/101 (42.6%)	61/167 (36.5%)	24/64 (37.5%)	0.86 (0.65–1.15)	0.81 (0.56–1.16)	-0.060 (−0.181 to 0.060)	−0.051 (−0.204 to 0.102)	17; 20

ANCS, antenatal corticosteroids; IPTW, inverse probability of treatment weighting; NEC, necrotizing enterocolitis; IVH, intraventricular hemorrhage; RDS, respiratory distress syndrome; RR, risk ratio; CI, confidence interval; composite outcome, mortality or severe NEC, IVH, or RDS; ARD, absolute risk difference; NNT, number needed to treat.

*^a^*Adjusted RRs were estimated using doubly robust modified Poisson models that incorporated stabilized IPTW trimmed to the 1st–99th percentiles and included gestational age at birth, birth weight, female sex, small-for-gestational-age status, plurality, pre-eclampsia, preterm premature rupture of membranes, clinical chorioamnionitis, nulliparity, and Induced delivery. Reference group = unexposed group.

*^b^*Result remains statistically significant after Holm step-down multiplicity adjustment (familywise α = 0.05) was applied to the four pre-specified secondary outcomes (severe RDS, severe NEC, severe IVH, and composite outcome). Adjusted two-sided 95% CIs are shown for all estimates.

*^c^*ARD was calculated as the risk in the treatment group minus the risk in the reference group (unexposed group). Negative values indicate risk reduction (benefit).

*^d^*NNT presented as ANCS-to-delivery interval ≤ 24 h; > 24 h–7 d vs. no exposure. NNT was calculated as 1/ARD when the ARD was statistically or clinically meaningful. NA = not applicable (ARD not clinically meaningful).

Multiplicity adjustment for four pre-specified secondary outcomes (severe RDS, NEC, IVH, and composite mortality or severe morbidity) used the Holm step-down procedure to control the family-wise error at 5%. Significance was assessed against multiplicity-adjusted α; results were reported as 95% CIs.

The robustness of primary results was assessed with four alternative approaches: (1) doubly robust OW, (2) IPTW with treatment-only Poisson model, (3) unweighted multivariable Poisson (covariates only), and (4) entropy-balancing weights with treatment-only Poisson model. Entropy balancing equalizes covariate means while minimizing the Kullback–Leibler distance and eliminating the need for further adjustments (Supplementary methods and Supplementary Table 5). The sensitivity analysis results (Tables 4–6 and Supplementary Table 6) were not adjusted for multiplicity and are reported with unadjusted 95% CIs.

**TABLE 4:** Sensitivity analysis: Association of first antenatal corticosteroid dose-to-delivery interval with neonatal outcomes (unweighted analysis).

Outcome	No exposure Events n/total (%)	ANCS-to-delivery interval ≤ 24 h Events n/total (%)	ANCS-to-delivery interval > 24 h–7 d Events n/total (%)	Crude RR ANCS-to-delivery interval ≤ 24 h	Crude RR ANCS-to-delivery interval > 24 h–7 d	Adjusted RR ^a^ ANCS-to-delivery interval ≤ 24 h	Adjusted RR ANCS-to-delivery interval > 24 h–7 d
Mortality	12/101 (11.9%)	11/167 (6.6%)	3/64 (4.7%)	0.55 (0.25–1.21)	0.39 (0.12–1.34)	0.58 (0.28–1.21)	0.35 (0.11–1.14)
Severe RDS	34/101 (33.7%)	55/167 (32.9%)	23/64 (35.9%)	0.98 (0.69–1.39)	1.07 (0.70–1.64)	0.98 (0.71–1.37)	1.00 (0.68–1.48)
Severe NEC	9/101 (8.9%)	3/167 (1.8%)	4/64 (6.2%)	0.20 (0.06–0.73)	0.70 (0.23–2.18)	0.15 (0.05–0.49)^b^	0.58 (0.18–1.87)
Severe IVH	4/101 (4.0%)	4/167 (2.4%)	2/64 (3.1%)	0.60 (0.15–2.37)	0.79 (0.15–4.18)	0.74 (0.18–3.13)	0.65 (0.13–3.30)
Composite outcome	43/101 (42.6%)	61/167 (36.5%)	24/64 (37.5%)	0.86 (0.63–1.16)	0.88 (0.60–1.30)	0.87 (0.66–1.15)	0.84 (0.59–1.19)
Sepsis^c^	40/94 (42.6%)	65/161 (40.4%)	22/62 (35.5%)	0.95 (0.70–1.28)	0.83 (0.55–1.26)	0.90 (0.69–1.18)	0.69 (0.47–1.01)

ANCS, antenatal corticosteroids; NEC, necrotizing enterocolitis; IVH, intraventricular hemorrhage; RDS, respiratory distress syndrome; RR, risk ratio; CI, confidence interval; composite outcome, either mortality or any severe morbidity (NEC, IVH, RDS). Crude RRs were obtained using an unadjusted modified Poisson regression (log link) with an HC3 robust variance.

*^a^*Adjusted RRs were obtained using modified Poisson regression, including gestational age at birth, birth weight, female sex, small-for-gestational-age status, plurality, pre-eclampsia, preterm premature rupture of membranes, clinical chorioamnionitis, nulliparity, and Induced delivery. Reference group = unexposed group.

*^b^*No multiplicity adjustment was applied. Family-wise error for the four pre-specified secondary outcomes was controlled in the primary inverse probability of treatment weighting analysis (Table 3).

*^c^*Sepsis was a *post hoc* safety outcome; no multiplicity adjustment was applied.

**TABLE 5:** Overlap-weighted, doubly robust sensitivity analysis: adjusted risk ratios for neonatal outcomes.

Outcome	No ANCS exposure Events n/Total (%)	ANCS-to-delivery interval ≤ 24 h Events n/Total (%)	ANCS-to-delivery interval > 24 h–7 d Events n/Total (%)	ANCS-to-delivery interval ≤ 24 h aRR^a^ (95% CI)	ANCS-to-delivery interval > 24 h–7 d aRR^a^ (95% CI)
Mortality	12/101 (11.9%)	11/167 (6.6%)	3/64 (4.7%)	0.52 (0.26–1.03)	0.35 (0.09–1.42)
Severe RDS	34/101 (33.7%)	55/167 (32.9%)	23/64 (35.9%)	0.96 (0.69–1.31)	0.98 (0.62–1.55)
Severe NEC	9/101 (8.9%)	3/167 (1.8%)	4/64 (6.2%)	0.11 ^b^ (0.03–0.38)	0.65 (0.15–2.73)
Severe IVH	4/101 (4.0%)	4/167 (2.4%)	2/64 (3.1%)	0.51 (0.15–1.76)	0.52 (0.07–3.77)
Composite outcome	43/101 (42.6%)	61/167 (36.5%)	24/64 (37.5%)	0.87 (0.66–1.14)	0.86 (0.57–1.29)

ANCS, antenatal corticosteroids; aRR, adjusted risk ratio; OW, overlap weighting; NEC, necrotizing enterocolitis; IVH, intraventricular hemorrhage; RDS, respiratory distress syndrome. Composite outcome = mortality or severe NEC, IVH, or RDS; Reference group = no ANCS.

*^a^*aRRs were obtained from modified Poisson regressions with HC3 robust variance, incorporating stabilized overlap weights and readjusting for 10 covariates (gestational age, birth weight, female sex, small-for-gestational-age status, twins, pre-eclampsia, preterm premature rupture of membranes, clinical chorioamnionitis, nulliparity, and Induced delivery). Each aRR group compared the indicated ANCS-to-delivery intervals with the no-ANCS reference group. CI, confidence interval.

*^b^*CI excludes 1. Effective sample sizes (ESS) by exposure group under OW were 98.5 (no ANCS), 160.5 (≤ 24 h), and 63.7(> 24 h–7 d) Outcomes were pre-specified; no multiplicity adjustment was applied. Confidence intervals (95%) are reported without correction and should be interpreted accordingly. Weighted totals may appear non-integer due to weighting.

**TABLE 6:** Entropy-balanced sensitivity analysis: adjusted risk ratios for neonatal outcomes.

Outcome	No ANCS exposure Events n/Total (%)	ANCS-to-delivery interval ≤ 24 h Events n/Total (%)	ANCS-to-delivery interval > 24 h–7 d Events n/Total (%)	ANCS-to-delivery interval ≤ 24 h aRR^a^ (95% CI)	ANCS-to-delivery interval > 24 h–7 d aRR ^a^ (95% CI)
Mortality	12/101 (11.9%)	11/167 (6.6%)	3/64 (4.7%)	0.62 (0.29–1.34)	0.38 (0.12–1.22)
Severe RDS	34/101 (33.7%)	55/167 (32.9%)	23/64 (35.9%)	0.93 (0.68–1.28)	0.95 (0.65–1.39)
Severe NEC	9/101 (8.9%)	3/167 (1.8%)	4/64 (6.2%)	0.19^b^ (0.06–0.62)	0.66 (0.20–2.14)
Severe IVH	4/101 (4.0%)	4/167 (2.4%)	2/64 (3.1%)	0.88 (0.21–3.72)	0.75 (0.16–3.52)
Composite outcome	43/101 (42.6%)	61/167 (36.5%)	24/64 (37.5%)	0.87 (0.66–1.15)	0.84 (0.59–1.19)

ANCS, antenatal corticosteroids; aRR, adjusted risk ratio; CI, confidence interval; NEC, necrotizing enterocolitis; IVH, intraventricular hemorrhage; RDS, respiratory distress syndrome; composite outcome = mortality or severe NEC, IVH, or RDS; Reference group (No ANCS).

*^a^*aRRs were obtained from a modified Poisson regression analysis with an HC3 robust variance, using entropy-balancing weights. No additional covariate adjustment was required because the weights achieved exact mean balance on the 10 pre-specified baseline variables (gestational age, birth weight, female sex, small-for-gestational-age status, plurality, pre-eclampsia, preterm premature rupture of membranes, clinical chorioamnionitis, nulliparity, and Induced preterm delivery). Each aRR group compared the specified ANCS-to-delivery interval with that of the reference group (No ANCS).

*^b^*CI excludes 1.

A pre-specified GA subgroup analysis (24–31 week) used the same 10-covariate modified Poisson model as the primary analysis, reporting aRRs with 95% CIs; no additional multiplicity adjustment was applied (Table 7). Extremely preterm births (< 28 weeks) were rare (40/332, 12%), precluding reliable stratum-specific estimates.

**TABLE 7:** Subgroup analysis: outcomes in infants 24–31 weeks by timing of first ANCS dose–to–delivery interval.

Outcome	No ANCS exposure events n/N	ANCS-to-delivery interval ≤ 24 h events n/N	ANCS-to-delivery interval > 24 h–7 d events n/N	ANCS-to-delivery interval ≤ 24 h aRR^a^	ANCS-to-delivery interval > 24 h–7 d aRR^a^
Mortality	8/51	9/92	3/42	0.64 (0.26–1.58)	0.43 (0.12–1.54)
Severe NEC	6/51	2/92	4/42	0.17^b^ (0.04–0.76)	0.87 (0.22–3.39)

ANCS, antenatal corticosteroid; aRR, adjusted risk ratio; 95% CI, confidence interval. Reference group: No ANCS.

*^a^*Adjusted risk ratios were estimated using modified Poisson regression with a log link and HC3 robust variance, adjusting for gestational age, birth weight, female sex, small-for-gestational-age status, plurality, pre-eclampsia, preterm premature rupture of membranes, clinical chorioamnionitis, nulliparity, and Induced preterm delivery.

*^b^*Outcomes were pre-specified; no multiplicity adjustment was applied to this subgroup analysis. Confidence intervals are unadjusted.

A *post hoc* exploratory economic analysis estimated hospital cost savings per severe NEC case prevented by combining unit cost data with weighted risk differences (Supplementary methods; Supplementary Table 7).

#### 2.7.1 Statistical software

Analyses were performed using R 4.3.0 (R Foundation for Statistical Computing, Vienna, Austria; Packages: *WeightIt, ebal, sandwich*). Two-sided *P* < 0.05, or 95% CIs excluding 1, were considered significant, with emphasis on effect estimates and CIs.

## 3 Results

### 3.1 Participant flow

**FIGURE 1:**
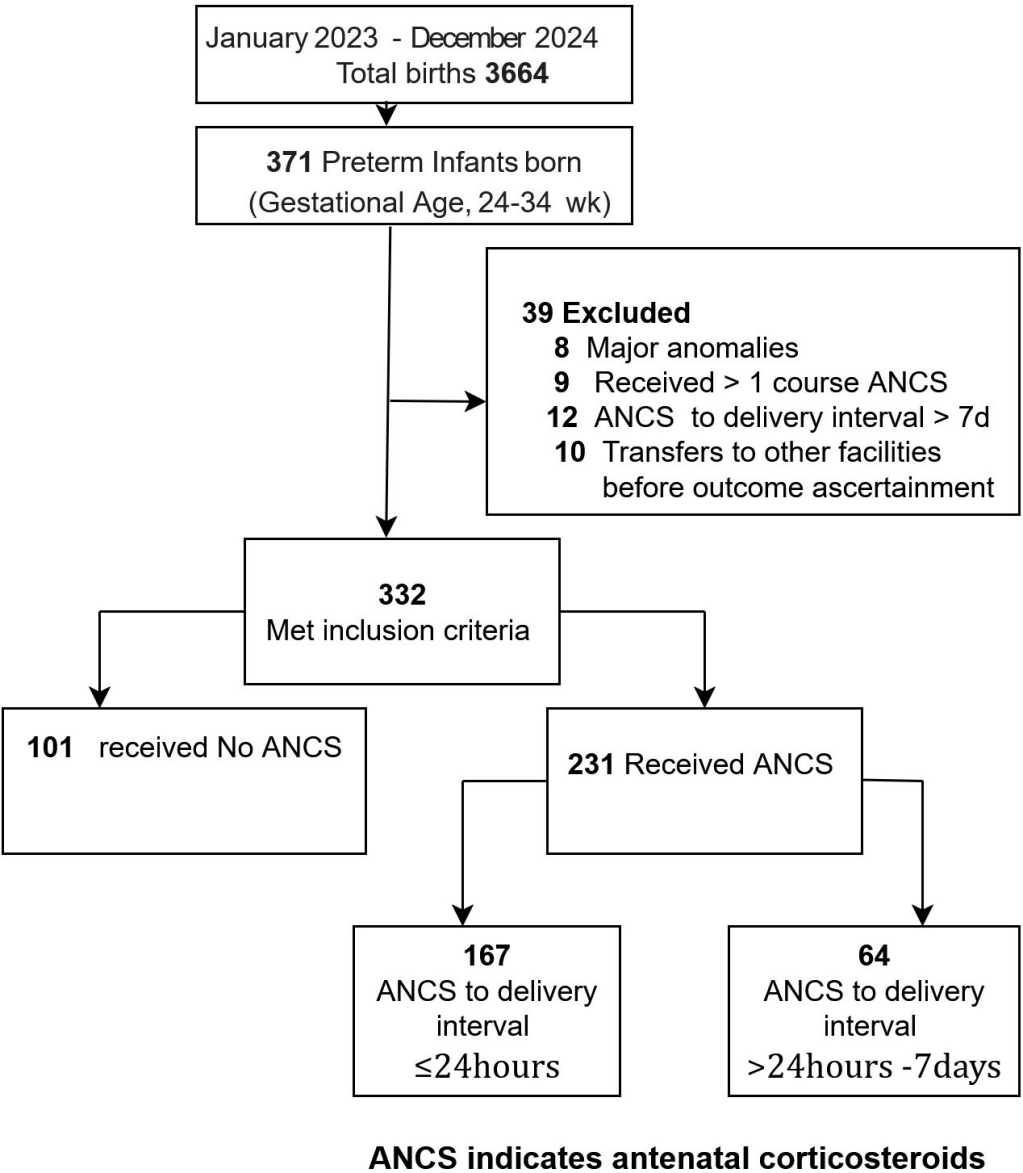
Participant flow diagram of the study. A total of 371 preterm infants were admitted to the neonatal intensive care unit during the study period. Of these, 39 were excluded because of major congenital anomalies (*n* = 8), receipt of > 1 course of antenatal corticosteroids (ANCS; *n* = 9), administration-to-delivery interval > 7 d (*n* = 12), or transfer before outcome ascertainment (*n* = 10). The final cohort comprised 332 infants. ANCS, antenatal corticosteroids

**FIGURE 2:**
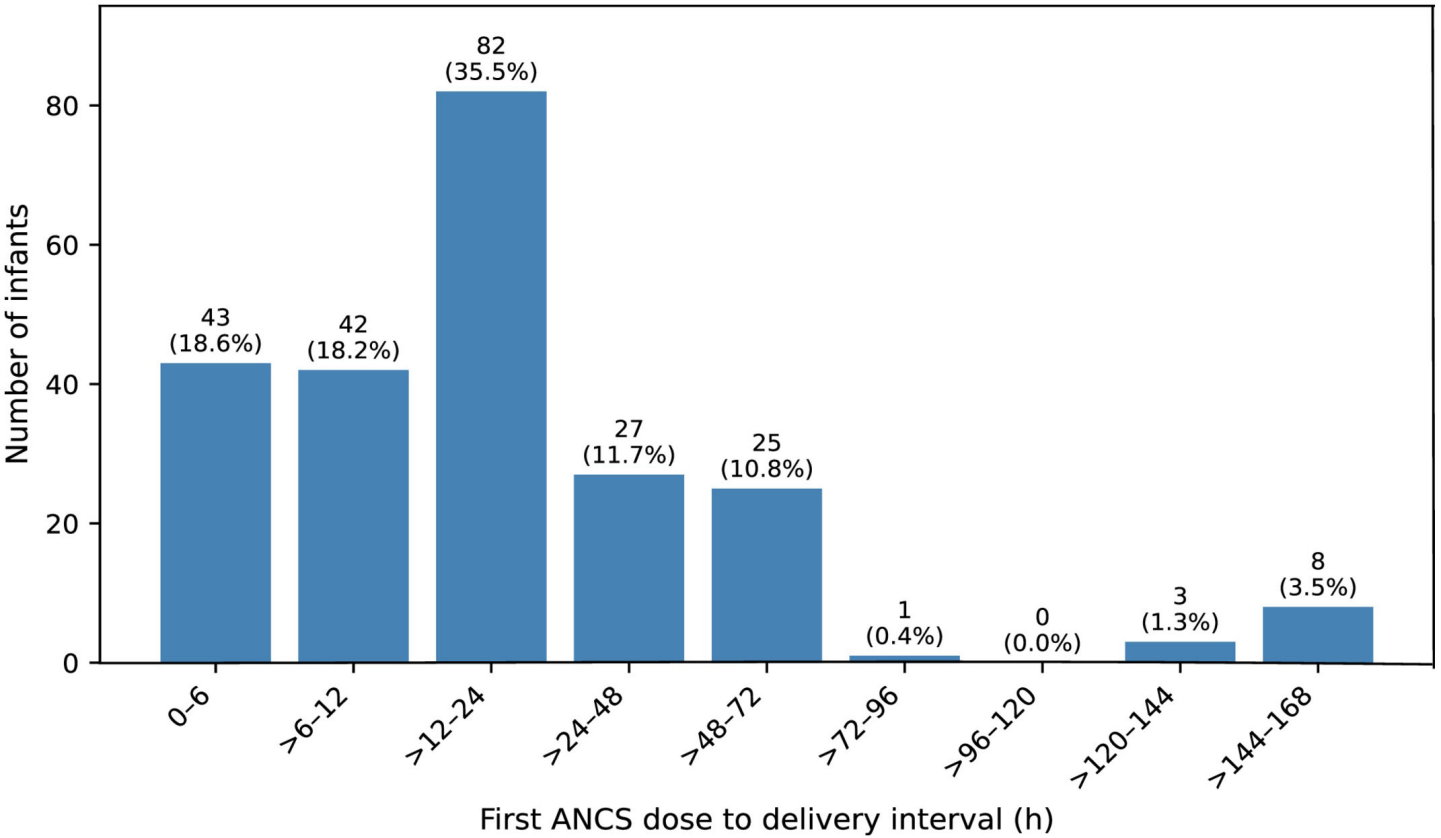
Distribution of preterm infants receiving the first antenatal corticosteroid dose to delivery interval in hours (*n* = 231). The bar graph shows the number and percentage (n [%]) of preterm infants who received antenatal corticosteroids at predefined intervals from the first dose to delivery. Most patients received ANCS within 12–24 h of delivery, with a peak in the > 12–24 h interval. ANCS, antenatal corticosteroid; h, hours.

Of the 371 preterm infants screened, 332 were enrolled (231 in the ANCS-exposed group and 101 in the unexposed group), exceeding the estimated sample size of 276 (Figure 1). The lower-than-expected mortality in the unexposed group (11.9% vs. 20% assumed) reduced the study’s statistical power. All ANCS-exposed infants received dexamethasone, with peak uptake occurring within the 12–24 h window (36% [82/231]) (Figure 2). The three exposure groups were broadly similar at baseline (Table 1). The mean (standard deviation) GA was 30.6 (2.3) week, and the mean birth weight was 1,428 (431) g. Of the 332 preterm deliveries, 230 (69.3%) were cesarean sections and 74 (22.3%) followed induced labor. Before weighting, the ANCS-exposed groups differed significantly from the unexposed group in 7 of 10 baseline covariates, with absolute SMDs > 0.20 (twin gestation and pre-eclampsia ≥ 0.27). Trimmed IPTW reduced these imbalances, with all covariates falling below the excellent balance threshold of 0.10. Overlap weighting also improved the balance, reducing all covariates to below 0.20, with three remaining above 0.10. Overall, weighting adequately aligned the baseline characteristics to permit unbiased outcome comparisons.

### 3.2 Primary analysis

Among the 332 very preterm infants, 7.8% (26/332) died: 6.6% (11/167) in the ≤ 24 h group, 4.7% (3/64) in the > 24 h–7 day dexamethasone-exposure group, and 11.9% (12/101) in the unexposed group. After inverse-probability-of-treatment weighting with covariate adjustment (doubly robust IPTW), neither exposure group differed significantly from the unexposed group (≤ 24 h: aRR, 0.58; 95% CI, 0.26–1.27; > 24 h–7 day: aRR, 0.33; 95% CI, 0.10–1.12; Table 3).

Severe NEC occurred in 1.8% of the ≤ 24 h exposure group and 8.9% of the unexposed group. Doubly robust IPTW confirmed a significant reduction (aRR, 0.15; 95% CI, 0.04–0.54). The > 24 h–7 d interval showed no significant effect (aRR, 0.52; 95% CI, 0.16–1.67). No other secondary outcomes differed significantly between the groups, and all CIs included 1 (Table 3).

### 3.3 Multiplicity adjustment

After Holm step-down correction for four pre-specified secondary outcomes, the reduction in severe NEC for the ≤ 24-h interval remained significant, while other adjusted outcomes were not significant (Table 3).

### 3.4 Sensitivity analyses

Multiple analytical approaches (Tables 4–6 and Supplementary Table 6) yielded consistent risk ratio estimates. All studies confirmed a reduction in the risk of NEC, with stable inferences across methods.

### 3.5 GA Subgroup analysis

Among infants born at 24–31 week of gestation (*n* = 185), aRRs were consistent with those in the primary analysis: mortality aRRs were 0.64 (95% CI, 0.26–1.58) in the ≤ 24 h exposure group and 0.43 (95% CI, 0.12–1.54) in the > 24 h–7 d exposure group; the aRR for severe NEC was 0.17 (95% CI, 0.04–0.76) in the ≤ 24 h exposure group (Table 7). The extremely preterm stratum (<28 week; *n* = 40) was not analyzed separately because of very small cell counts that would yield unstable estimates.

### 3.6 *Post hoc* economic and safety implications

*Post hoc* exploratory cost analysis estimated savings of approximately $4,600 per severe NEC case averted (Table 3 and Supplementary Table 7). Dexamethasone exposure did not increase the risk of maternal chorioamnionitis or sepsis (Tables 1, 4). These findings support a meaningful reduction in severe NEC when dexamethasone is administered ≤ 24 h before delivery, with no detectable effects on mortality or other severe neonatal morbidities.

## 4 Discussion

We evaluated the association between ANCS administration timing and neonatal outcomes in preterm infants born at 24–34 week of gestation. Dexamethasone administered within 24 h before delivery was associated with a significant reduction in severe NEC compared to no dexamethasone administration; however, there was no measurable benefit in terms of mortality or other severe morbidities.

Mortality was lower in the ≤ 24 h exposure group than in the unexposed group (6.6% vs. 11.9%); however, the confidence interval was wide (aRR, 0.58; 95% CI, 0.26–1.27), indicating insufficient power to confirm a survival benefit. Although the target enrollment was achieved, the lower-than-expected mortality rate in the dexamethasone-unexposed group (11.9% vs. the 20% assumed in *a priori* power calculations) reduced statistical power. This highlights a common limitation of single-center observational research, wherein variation in baseline event rates can hinder adequate power for mortality outcomes. Therefore, these findings should be interpreted with caution and considered hypothesis-generating.

Previous large cohort studies have reported inconsistent results. Two studies (15, 16) observed early mortality benefits within hours of betamethasone administration in extremely preterm infants, whereas a secondary analysis of data from the WHO ACTION-I cohort, which included moderately preterm infants in LMICs, found non-significant trends toward reduced mortality within 24 h of dexamethasone administration (17). These discrepancies likely reflect differences in the study populations, ANCS formulations, and care settings. Larger, adequately powered, multicenter studies are needed to clarify the effects of ANCS administration timing on LMICs.

### 4.1 Secondary outcomes: severe NEC

Severe NEC within 24 h of dexamethasone exposure was reduced by 85% compared with no exposure (1.8% vs. 8.9%; aRR, 0.15; 95% CI, 0.04–0.54). This association remained significant after adjusting for multiple comparisons and was consistent across the sensitivity analyses, supporting the robustness of the findings. No other secondary outcomes differed significantly between the groups.

The reduction in NEC incidence is clinically important. NEC affects 5–10% of very low birth weight infants, with mortality rates of 20–30%. In LMICs, delayed diagnosis and limited surgical capacity exacerbate outcomes (23, 24). Even a modest decrease in NEC incidence can yield substantial clinical and economic benefits (Supplementary Table 7).

Evidence regarding the optimal timing of ANCS administration for NEC prevention remains inconclusive. Some studies have reported either no benefit or an increased risk associated with very short exposure intervals (25–27). In contrast, the EPICE cohort of very preterm infants (14) and Chawla et al. (15) reported reductions in NEC within hours of betamethasone administration in extremely preterm infants. In our pre-specified sensitivity analysis of infants born at 24–31 week of gestation, results aligned with those of the EPICE study, demonstrating a significant reduction in severe NEC when dexamethasone was administered within 24 h before delivery. The observed benefit, despite a higher mean GA (31 week), suggests a potential protective effect that warrants further investigation. Given its wider availability and lower cost compared to betamethasone, dexamethasone may be particularly valuable in LMIC settings (28, 29).

### 4.2 Mechanistic considerations

The reduction in severe NEC with brief ANCS exposure is plausibly mediated by rapid, non-genomic glucocorticoid actions through membrane-bound receptors and secondary messenger pathways, including protein kinase A and protein kinase C. These pathways can rapidly downregulate inflammatory cascades (e.g., nuclear factor-κB signaling), decrease pro-inflammatory cytokine release, and enhance epithelial barrier integrity within minutes, potentially limiting mucosal injury in the immature gut (18, 23). However, these mechanisms remain theoretical and require further experimental confirmation. ANCS administration within 24 h has been shown to protect against severe RDS (25). However, given the mean GA of 31 week in this cohort, pulmonary maturity and substantial endogenous surfactant production (30) may have attenuated the potential benefit for RDS prevention. Contemporary NICU practices, including the liberal use of exogenous surfactants and non-invasive respiratory support, may further obscure the effects of ANCS administration timing on pulmonary outcomes. Additionally, inconsistencies in RDS definitions and variability across studies examining ANCS administration timing have led to the exclusion of RDS from composite endpoints in several cohorts (16).

### 4.3 Safety profile and economic implications

Dexamethasone administration within 24 h before delivery did not increase the risk of chorioamnionitis or neonatal sepsis, supporting its safety and alignment with previous reports (31). A *post hoc* exploratory cost analysis (Supplementary Table 7) estimated savings of approximately $4,600 per severe NEC case averted, consistent with the cost data from Indian neonatal care (32, 33). However, the exploratory design, reliance on local cost estimates, and potential confounding factors limit generalizability and underscore the need for prospective studies with pre-specified economic endpoints.

### 4.4 Study strengths and limitations

The prospective design, standardized outcome definitions, and robust propensity score methods with IPTW helped reduce confounding. Sensitivity analyses using doubly robust overlap weighting and entropy balancing yielded similar estimates, strengthening confidence in the findings. As this study was conducted in a tertiary NICU in India, it provides valuable evidence from an LMIC setting that remains underrepresented in research on the timing of ANCS administration.

This study has several limitations that warrant consideration. Although the design mitigated key biases, as an observational, single-center study, residual confounding cannot be excluded, particularly from urgent deliveries clustering in shorter ANCS-to-delivery intervals, a bias that would likely attenuate the observed benefits and thereby strengthen our conclusions. Additional unmeasured confounders, such as socioeconomic status and travel time, may remain. Important unmeasured NEC-related factors include feeding protocols, antibiotic regimens, central catheter practices, and surgical policies; however, the substantial risk reduction (absolute risk difference = -7.1%, number needed to treat = 14) after multiplicity adjustment supports the robustness of our findings.

The lower-than-expected mortality in the unexposed group (11.9% vs. 20%) reduced the statistical power for the primary outcome. The small sample size and low event rates necessitated categorical rather than continuous modeling of ANCS-to-delivery intervals (14–17), precluding detailed temporal analysis. Limited representation of the extremely preterm subgroup (< 28 week; 12%) and the tertiary NICU setting may limit generalizability to this subpopulation and to typical LMIC contexts, particularly rural or secondary facilities, where most births in India occur (13). Physiologic risks vary across 24–34 week of gestation; our models adjusted for GA and achieved excellent post-weighting covariate balance to mitigate this heterogeneity. The 24–31 week sensitivity analysis yielded materially consistent estimates, supporting robustness within earlier gestations.

Our exposure definition (first-dose-to-delivery interval) was chosen to reflect the onset of steroid action and to minimize bias associated with last-dose timing proximal to delivery. Although delivery mode and urgency were not explicitly modeled as primary covariates, adjustment for upstream clinical indications (Induced preterm delivery, PPROM, pre-eclampsia) addressed confounding by indication related to delivery mode and urgency, as these factors determine whether cesarean section, induction, or spontaneous vaginal delivery occurs.

### 4.5 Clinical implications and future directions

These hypothesis-generating findings do not warrant changes to current ANCS administration timing guidelines, which recommend dexamethasone use at 24–34 week of gestation without specifying optimal intervals (6, 7). The 2021 FIGO recommendations advise administration “ideally 18–72 h before preterm birth is expected,” but explicitly state that “if preterm birth is expected within 18 h, prenatal corticosteroids should still be administered” (10). Our finding of a significant reduction in NEC with ≤ 24 h exposure provides empirical support for administering ANCS even when delivery is imminent, particularly in LMIC settings where most women present late in labor and the ideal timing window cannot consistently be achieved. The observed reduction in severe NEC risk is clinically meaningful; however, the observational, single-center design and potential residual confounding necessitate cautious interpretation.

Future research should prioritize multicenter, adequately powered trials, such as cluster-randomized designs, to compare ANCS administration timing strategies in low-resource settings. Mechanistic studies exploring the effects of ANCS on gut development and inflammation may clarify the observed NEC findings. Implementation research should address barriers to timely ANCS delivery in LMICs, and long-term neurodevelopmental, metabolic, and cost-effectiveness outcomes should also be evaluated.

## 5 Conclusion

Maternal dexamethasone administered within 24 h before preterm birth may reduce the risk of severe NEC but has no confirmed survival benefit. Imprecise estimates of the primary mortality outcome and the potential for residual confounding preclude definitive conclusions regarding the optimal timing of ANCS administration. These findings underscore the need for larger, adequately powered trials in diverse settings, including rural and secondary facilities, to address the variability in ANCS uptake and resource availability in LMICs. ANCS should continue to be offered to all eligible women, even when only brief intervals before delivery are available, to ensure the completion of a single course.

## Data Availability

The raw data supporting the conclusions of this article will be made available by the authors, without undue reservation.

## References

[1] Bradley E Blencowe H Moller A Okwaraji Y Sadler F Gruending A Born too soon: global epidemiology of preterm birth and drivers for change. *Reprod Health.* (2025) 22:105. 10.1186/s12978-025-02033-x 40551192 PMC12186353

[2] Sankar M Neogi S Sharma J Chauhan M Srivastava R Prabhakar P State of newborn health in India. *J Perinatol.* (2016) 36:S3–8. 10.1038/jp.2016.183 27924104 PMC5144119

[3] Liggins G Howie RN. A controlled trial of antepartum glucocorticoid treatment for prevention of the respiratory distress syndrome in premature infants. *Pediatrics.* (1972) 50:515–25. 10.1542/peds.50.4.515 4561295

[4] Roberts D Brown J Medley N Dalziel S. Antenatal corticosteroids for accelerating fetal lung maturation for women at risk of preterm birth. *Cochrane Database Syst Rev.* (2017) 3:CD004454. 10.1002/14651858.CD004454.pub3 28321847 PMC6464568

[5] McGoldrick E Stewart F Parker R Dalziel S. Antenatal corticosteroids for accelerating fetal lung maturation for women at risk of preterm birth. *Cochrane Database Syst Rev.* (2020) 12:CD004454. 10.1002/14651858.CD004454.pub4 33368142 PMC8094626

[6] Committee on Obstetric Practice. Committee opinion No. 713: antenatal corticosteroid therapy for fetal maturation. *Obstet Gynecol.* (2017) 130:e102–9. 10.1097/AOG.0000000000002237 28742678

[7] Vogel J Ramson J Darmstadt G Qureshi Z Chou D Bahl R Updated WHO recommendations on antenatal corticosteroids and tocolytic therapy for improving preterm birth outcomes. *Lancet Glob Health.* (2022) 10:e1707–8. 10.1016/S2214-109X(22)00434-X 36400080 PMC9681658

[8] Melamed N Shah J Soraisham A Yoon E Lee S Shah P Association between antenatal corticosteroid administration-to-birth interval and outcomes of preterm neonates. *Obstet Gynecol.* (2015) 125:1377–84. 10.1097/AOG.0000000000000840 26000509

[9] Norberg H Kowalski J Marsal K Norman M. Timing of antenatal corticosteroid administration and survival in extremely preterm infants: a national population-based cohort study. *BJOG.* (2017) 124:1567–74. 10.1111/1471-0528.14545 28294496

[10] Norman J Shennan A Jacobsson B Stock S FIGO Working Group for Preterm Birth. FIGO good practice recommendations on the use of prenatal corticosteroids to improve outcomes and minimize harm in babies born preterm. *Int J Gynaecol Obstet.* (2021) 155:26–30. 10.1002/ijgo.13836 34520057

[11] McDougall A Aboud L Lavin T Cao J Dore G Ramson J Effect of antenatal corticosteroid administration-to-birth interval on maternal and newborn outcomes: a systematic review. *EClinicalMedicine.* (2023) 58:101916. 10.1016/j.eclinm.2023.101916 37007738 PMC10050784

[12] Vogel J Oladapo O Pileggi-Castro C Adejuyigbe E Althabe F Ariff S Antenatal corticosteroids for women at risk of imminent preterm birth in low-resource countries: the case for equipoise and the need for efficacy trials. *BMJ Glob Health.* (2017) 2:e000398. 10.1136/bmjgh-2017-000398 29082019 PMC5656119

[13] Kankaria A Duggal M Chauhan A Sarkar D Dalpath S Kumar A Readiness to provide antenatal corticosteroids for threatened preterm birth in public health facilities in Northern India. *Glob Health Sci Pract.* (2021) 9:575–89. 10.9745/GHSP-D-20-00716 34593583 PMC8514043

[14] Norman M Piedvache A Børch K Huusom L Bonamy A Howell E Association of short antenatal corticosteroid administration-to-birth intervals with survival and morbidity among very preterm infants: results from the EPICE cohort. *JAMA Pediatr.* (2017) 171:678–86. 10.1001/jamapediatrics.2017.0602 28505223 PMC5710338

[15] Chawla S Wyckoff M Lakshminrusimha S Rysavy M Patel R Chowdhury D Short duration of antenatal corticosteroid exposure and outcomes in extremely preterm infants. *JAMA Netw Open.* (2025) 8:e2461312. 10.1001/jamanetworkopen.2024.61312 39982720 PMC11846007

[16] Melamed N Murphy K Pylypjuk C Sherlock R Ethier G Yoon E Timing of antenatal corticosteroid administration and neonatal outcomes. *JAMA Netw Open.* (2025) 8:e2511315. 10.1001/jamanetworkopen.2025.11315 40388165 PMC12090034

[17] WHO ACTION Trials Collaborators. Effect of dexamethasone on newborn survival at different administration-to-birth intervals: a secondary analysis of the WHO ACTION (Antenatal CorticosTeroids for improving outcomes in preterm newborn)-I trial. *EClinicalMedicine.* (2022) 53:101744. 10.1016/j.eclinm.2022.101744 36467459 PMC9716334

[18] Panettieri R Schaafsma D Amrani Y Koziol-White C Ostrom R Tliba O. Non-genomic effects of glucocorticoids: an updated view. *Trends Pharmacol Sci.* (2019) 40:38–49. 10.1016/j.tips.2018.11.002 30497693 PMC7106476

[19] Ministry of Health & Family Welfare. *India’s Newborn Action Plan (INAP).* (2014). Available online at: https://nhm.gov.in/images/pdf/programmes/inap-final.pdf (accessed July 24, 2025).

[20] Li F Morgan K Zaslavsky A. Balancing covariates via propensity score weighting. *J Am Stat Assoc.* (2017) 113:390–400. 10.1080/01621459.2016.1260466

[21] Rubin D. Using propensity scores to help design observational studies: application to the tobacco litigation. *Health Serv Outcomes Res Methodol.* (2001) 2:169–88. 10.1023/a:1020363010465

[22] Funk M Westreich D Wiesen C Sturmer T Brookhart M Davidian M. Doubly robust estimation of causal effects. *Am J Epidemiol.* (2011) 173:761–7. 10.1093/aje/kwq439 21385832 PMC3070495

[23] Deshpande G Jape G Rao S Patole S. Benefits of probiotics in preterm neonates in low-income and medium-income countries: a systematic review of randomised controlled trials. *BMJ Open.* (2017) 7:e017638. 10.1136/bmjopen-2017-017638 29222137 PMC5728295

[24] Singh J Sinha S. Necrotizing enterocolitis—an unconquered disease. *Indian Pediatr.* (2002) 39:229–37. 11910131

[25] Siegler Y Justman N Bachar G Lauterbach R Zipori Y Khatib N Is there a benefit of antenatal corticosteroid when given < 48 h before delivery? *Arch Gynecol Obstet.* (2022) 306:1463–8. 10.1007/s00404-022-06411-9 35099594

[26] Borgida A DeGroff S Fuller K. Neonatal outcomes based on antenatal corticosteroid exposure time for infants delivered between 23 and 34 weeks gestation. *Clin Exp Obstet Gynecol.* (2017) 44:247–51. 10.12891/ceog3469.2017 29746032

[27] Battarbee A Ros S Esplin M Biggio J Bukowski R Parry S Optimal timing of antenatal corticosteroid administration and preterm neonatal and early childhood outcomes. *Am J Obstet Gynecol MFM.* (2020) 2:100077. 10.1016/j.ajogmf.2019.100077 32905377 PMC7469940

[28] WHO ACTION Trial Collaborators. Antenatal dexamethasone for improving preterm newborn outcomes in low-resource countries: a cost-effectiveness analysis of the WHO ACTION-I trial. *Lancet Glob Health.* (2022) 10:e1523–33. 10.1016/S2214-109X(22)00340-0 36113535

[29] Williams M Ramson J Brownfoot F. Different corticosteroids and regimens for accelerating fetal lung maturation for babies at risk of preterm birth. *Cochrane Database Syst Rev.* (2022) 8:CD006764. 10.1002/14651858.CD006764.pub4 35943347 PMC9362990

[30] Liu S Yang H Chen C Chou H Hsieh W Tsou K The gestational effect of antenatal corticosteroids on respiratory distress syndrome in very low birth weight infants: a population-based study. *J Formos Med Assoc.* (2020) 119:1267–73. 10.1016/j.jfma.2019.11.002 31761503

[31] Travers C Carlo W McDonald S Das A Bell E Ambalavanan N Mortality and pulmonary outcomes of extremely preterm infants exposed to antenatal corticosteroids. *Am J Obstet Gynecol.* (2018) 218:130.e1–13. 10.1016/j.ajog.2017.11.554 29138031 PMC5842434

[32] Narang A Kiran P Kumar P. Cost of neonatal intensive care in a tertiary care center. *Indian Pediatr.* (2005) 42:989–97. 16269829

[33] Prinja S Manchanda N Mohan P. Cost of neonatal intensive care delivered through district level public hospitals in India. *Indian Pediatr.* (2013) 50:839–46. 10.1007/s13312-013-0234-y 23502671

