# Supplementary material for: Association of antenatal dexamethasone administration timing with outcomes in preterm infants in a low- and middle-income country
Source: Front Med (Lausanne). 2025 Nov 27;12:1712437. doi: 10.3389/fmed.2025.1712437 (PMC12695745; doi:10.3389/fmed.2025.1712437)
Supplement: Supplementary file 1 [file Supplementary_file_1.docx]

**Supplementary Material**

1. **Supplementary** **Methods**
2. Additional sensitivity analysis (Entropy balancing weights)
   1. Exploratory Post-hoc economic analysis
3. **Supplementary** **Tables**

Supplementary Table S1. Baseline characteristics and covariate balance before and after weighting

Supplementary Table S2. Baseline characteristics after stabilized-trimmed inverse probability of treatment weighting

Supplementary Table S3. Baseline characteristics after overlap weighting

Supplementary Table S4. Stability diagnostics for stabilized propensity-score weighting.

Supplementary Table S5. Entropy-balancing diagnostics: standardized mean differences before and after weighting

Supplementary Table S6. Inverse probability of treatment weighted sensitivity analysis: : Adjusted risk ratios for neonatal outcomes .

Supplementary Table S7. Exploratory economic evaluation assumptions

1. **Supplementary** **Methods**

**1.1 Additional sensitivity analysis (Entropy balancing weights)**

Rationale and implementation: To verify that the modeling choices did not drive our findings, we re-estimated the treatment effects using entropy-balancing weights, an alternative, fully non-parametric approach that forces the weighted mean of every baseline covariate to match the corresponding mean in the combined sample. Separate weights were generated for each exposure group (no ANCS, ANCS ≤24 h, ANCS >24 h–7 d). Because the moment-matching constraints are solved to numerical precision, covariate imbalance is removed by design, eliminating the need for further regression adjustments. Therefore, the weighted analyses used a treatment-only modified Poisson model.

**1.2 Entropy balance diagnostics ( Supplementary Table S5)**

Before weighting, several covariates showed a moderate imbalance (e.g., plurality, SMD = 0.27). Entropy balancing reduced every absolute SMD to 0.00, achieving perfect mean balance across all 10 pre-specified characteristics, well within the conventional SMD threshold of <0.10.

**1.3 Exploratory post-hoc economic analysis**

Following the completion of the clinical analyses, we conducted an unplanned exploratory cost-effectiveness evaluation focusing on severe necrotizing enterocolitis (NEC). Using the IPTW doubly robust model (Table 2, main manuscript), we calculated the risk difference for NEC in the ANCS ≤24 h group and converted it to the number needed to treat (NNT = 14, corresponding to a 7.1% absolute risk reduction).

**1.3.1 Cost inputs.**

Direct medical costs were adapted from the 2024 price lists at two tertiary hospitals in northern India: INR 50–100 per antenatal corticosteroid course and INR 400,000 per NEC episode. (1) Costs are expressed in USD, using the mid-2025 exchange rate (1 USD = INR 86.6).

**1.3.2 Health benefits.**

Benefits were expressed as disability-adjusted life-years (DALYs) averted, combining (1) years of life lost under alternative NEC mortalities (10–30%) and (2) years lived with disability for survivors (weight 0.20–0.30, duration 10–20 years). One-way sensitivity analyses varied the parameters across ranges.(1-4) All economic inputs are detailed in Supplementary Table S7.

**2 Supplementary Results — Economic implications (Supplementary Table S7)**

Applying an NNT of 14, preventing one NEC case would require ANCS drug costs of approximately INR 700–1,400 (≈US$8–16) versus expected treatment costs of INR 400,000 (≈US$4,620). Therefore, the net saving is ≈INR 398,600–399,300 (US$4,604–$4,612) per NEC case averted.

Depending on mortality and disability assumptions, each NEC case prevented translation to 5–10 DALYs averted, yielding an implied cost of <US$1,000 per DALY saved, well within conventional cost-effectiveness thresholds for low- and middle-income countries and comparable to WHO ACTION-I estimates (US$400–800 per DALY).(5)

These findings suggest that short-interval ANCS exposure is economically attractive in resource-limited settings. However, the analysis was *post-hoc*, used local cost data, and was subject to residual confounding factors. The confirmation of prospectively designed studies with pre-specified economic endpoints is warranted.

1. **Supplementary** **Tables**

**Supplementary Table S1. Baseline characteristics and covariate balance before and after weighting**

| **Covariate** | **No ANCS exposure** | **ANCS to delivery interval ≤24 h** | **ANCS to delivery interval >24 h–7 d** | **P value** | **UnW_SMD for ANCS-to-delivery interval ≤24 h** | **UnW_SMD for ANCS-to-delivery interval >24 h** | **IPTW SMD for ANCS-to-delivery interval ≤24 h** | **IPTW SMD for ANCS-to-delivery interval>24 h** | **OW SMD for ANCS-to-delivery interval ≤24 h** | **OW SMD for ANCS-to-delivery interval >24 h** |  |
| --- | --- | --- | --- | --- | --- | --- | --- | --- | --- | --- | --- |
| Gestational age, mean SD, wk | 30.7 (2.4) | 30.7 (2.1) | 30.2 (2.1) | 0.378 | 0.01 | 0.19 | 0.01 | 0.04 | 0.01 | 0.14 |  |
| Birth weight mean SD, g | 1,440.8 (429.0) | 1,429.5 (425.3) | 1,405.5 (456.0) | 0.877 | 0.03 | 0.08 | 0.01 | 0.03 | 0.02 | 0.07 |  |
| Female sex | 44 (43.6%) | 66 (39.5%) | 21 (32.8%) | 0.387 | 0.08 | 0.22 | 0.01 | 0.01 | 0.13 | 0.26 |  |
| SGA | 9 (8.9%) | 28 (16.8%) | 8 (12.5%) | 0.184 | 0.24 | 0.12 | 0.05 | 0.05 | 0.17 | 0.09 |  |
| Twin Pregnancy | 18 (17.8%) | 49 (29.3%) | 16 (25.0%) | 0.108 | 0.27 | 0.18 | 0.07 | 0.05 | 0.28 | 0.18 |  |
| Pre-eclampsia | 22 (21.8%) | 57 (34.1%) | 18 (28.1%) | 0.096 | 0.28 | 0.15 | 0.06 | 0.06 | 0.23 | 0.15 |  |
| PPROM | 20 (19.8%) | 41 (24.6%) | 17 (26.6%) | 0.547 | 0.11 | 0.16 | 0.02 | 0.02 | 0.13 | 0.14 |  |
| Clinical chorioamnionitis | 1 (1.0%) | 5 (3.0%) | 3 (4.7%) | 0.344 | 0.14 | 0.22 | 0.12 | 0.15 | 0.1 | 0.2 |  |
| Nulliparity | 51 (50.5%) | 88 (52.7%) | 30 (46.9%) | 0.727 | 0.04 | 0.07 | 0.04 | 0.04 | 0.0 | 0.11 |  |
| Induced preterm delivery | 19 (18.8%) | 39 (23.4%) | 16 (25.0%) | 0.581 | 0.11 | 0.15 | 0.05 | 0.01 | 0.14 | 0.15 |  |

Abbreviations: ANCS, antenatal corticosteroids; SMD, absolute standardized mean difference; UnW, unweighted; IPTW, inverse probability of treatment weighting; OW, overlap weighting; PROM, preterm premature rupture of membranes; SGA, small for gestational age. Continuous variables are presented as mean (SD), and categorical variables are presented as numbers (percentages). SMDs compared each treated group with the no-ANCS group; an SMD < 0.10 denotes adequate balance. P-values from one-way ANOVA (continuous) or χ^2^ tests (categorical). weights derived from multinomial logistic model with HC1 robust variance

**Supplementary Table S2 . Baseline characteristics after stabilized-trimmed inverse probability of treatment weighting**

| **Variable** | **Maximum abs SMD** | **No ANCS^a^**  **exposure** | **ANCS-to-delivery interval ≤ 24 h^a^** | **ANCS-to-delivery interval > 24 h–7 d^a^** |
| --- | --- | --- | --- | --- |
| Gestational age, mean SD, wk | 0.04 | 30.61 (2.44) | 30.6 (2.19) | 30.52 (2.02) |
| Birth weight mean SD, g | 0.03 | 1434.17 (432.79) | 1431.27 (423.78) | 1420.11 (450.27) |
| Female sex | 0.01 | 40.4 (39.7%) | 66.8 (40.0%) | 24.8 (39.4%) |
| SGA | 0.03 | 12.2 (12.0%) | 21.9 (13.1%) | 8.1 (12.9%) |
| Twin pregnancy | 0.08 | 29.4 (28.9%) | 42.3 (25.3%) | 16.6 (26.4%) |
| Pre-eclampsia | 0.08 | 32.9 (32.3%) | 48.9 (29.2%) | 18.0 (28.6%) |
| PPROM | 0.01 | 23.6 (23.2%) | 39.7 (23.7%) | 14.8 (23.6%) |
| Clinical chorioamnionitis | 0.1 | 1.7 (1.6%) | 4.7 (2.8%) | 2.0 (3.1%) |
| Nulliparity | 0.04 | 49.7 (48.8%) | 84.1 (50.3%) | 31.9 (50.7%) |
| Induced preterm delivery | 0.05 | 25.7 (25.2%) | 38.6 (23.1%) | 15.5 (24.7%) |

**Abbreviations**: ANCS, antenatal corticosteroids abs SMD, absolute standardized mean difference; PPROM, preterm pre-labor rupture of membranes; SGA, small-for-gestational age. Continuous covariates shown as mean (SD); binary as weighted n (weighted %). abs SMDs represent the largest of the three pair-wise contrasts among exposure groups (No ANCS vs. ≤ 24 h, No ANCS vs. > 24 h–7 d, and ≤ 24 h vs. > 24 h–7 d); |SMD| < 0.10 indicates excellent balance.

Propensity scores were estimated using a multinomial logistic regression (statsmodels MNLogit) with HC1 robust variance; class probabilities were obtained via the softmax of the fitted coefficients. Stabilized IPTW with 1st–99th percentile trimming, mean-rescaling to 1.0, and capping at 20 was applied.

Effective sample sizes (ESS) by exposure group under IPTW were 85.9 (no ANCS), 161.3 (≤ 24 h), and 60.0.(> 24 h–7 d)

**^a^**Counts are weight-adjusted and may appear as non-integer values.

**Supplementary Table S3. Baseline characteristics after overlap weighting**

| **Variable** | **Maximum abs SMD** | **No ANCS exposure ^a^** | **ANCS-to-delivery interval ≤ 24 h^a^** | **ANCS-to-delivery interval > 24 h–7 d^a^** |
| --- | --- | --- | --- | --- |
| Birth weight in grams | 0.07 | 1440.69 (431.73) | 1433.95 (423.12) | 1409.47 (453.52) |
| Clinical chorioamnionitis | 0.17 | 1.4 (1.2%) | 3.8 (2.8%) | 3.4 (4.0%) |
| Gestational age in weeks | 0.14 | 30.65 (2.44) | 30.59 (2.19) | 30.32 (2.09) |
| Nulliparity | 0.05 | 56.3 (50.3%) | 68.2 (50.5%) | 40.9 (48.1%) |
| PPROM | 0.09 | 24.2 (21.6%) | 32.5 (24.0%) | 21.8 (25.6%) |
| Pre-eclampsia | 0.07 | 28.9 (25.8%) | 38.7 (28.7%) | 24.2 (28.4%) |
| Female sex | 0.16 | 47.0 (42.0%) | 53.5 (39.7%) | 29.2 (34.2%) |
| Twin pregnancy | 0.09 | 24.1 (21.6%) | 33.5 (24.8%) | 21.6 (25.4%) |
| SGA | 0.08 | 11.2 (10.0%) | 16.5 (12.2%) | 10.8 (12.7%) |
| Induced preterm delivery | 0.1 | 23.4 (20.9%) | 31.2 (23.1%) | 21.3 (25.0%) |

**Abbreviations**: ANCS, antenatal corticosteroids abs SMD, absolute standardized mean difference; OW, overlap weighting; PPROM, preterm pre-labor rupture of membranes; SGA, small-for-gestational age. continuous shown as mean (SD); binary as weighted n (weighted %). abs SMDs represent the largest of the three pair-wise contrasts among exposure groups (No ANCS vs. ≤ 24 h, No ANCS vs. > 24 h–7 d, and ≤ 24 h vs. > 24 h–7 d); |SMD| < 0.10 denotes excellent balance. Propensity scores were estimated using a multinomial logistic regression (statsmodels MNLogit) with HC1 robust variance; class probabilities were obtained via the softmax of the fitted coefficients. Overlap weights were defined as 1 – p observed for each individual.

Effective sample sizes (ESS) by exposure group under OW were 98.5 (no ANCS), 160.5 (≤ 24 h), and 63.7(> 24 h–7 d)

**^a^**Counts are weight-adjusted and may appear as non-integer values.

**Supplementary Table S4. Stability diagnostics for stabilized propensity-score weighting**

| **Statistic** | **IPTW** | **OW** | **EBal** |
| --- | --- | --- | --- |
| Effective sample size (N) | 306.08 | 311.02 | 289.37 |
| Minimum weight | 0.59 | 0.23 | 0.29 |
| Median | 0.95 | 0.58 | 1.000 |
| Mean | 1.00 | 0.59 | 1.000 |
| Maximum weight | 2.19 | 0.90 | 4.73 |
| Proportion of weights >5 | 0.00 | 0.00 | 0.000 |
| Proportion of weights >10 | 0.00 | 0.00 | 0.000 |
| Proportion of weights >20 | 0.00 | 0.00 | 0.000 |

**Abbreviations**: IPTW, inverse probability of treatment weighting; OW, overlap weighting; EBal, entropy balancing; N, sample size. IPTW used stabilized weights with 1st to 99th percentile trimming, mean rescaled to 1.0, and capping at 20. OW was defined as 1 minus p_observed from a multinomial logistic regression (statsmodels MNLogit) with HC1 robust variance; class probabilities were obtained via softmax. Stability metrics summarize the final analysis weights. Effective sample size was computed as (sample size (Σw)^2^/Σw^2^ was close to the raw cohort size (n = 332), suggesting minimal precision loss. A mean or median near 1, or a median near 0, and the absence of extreme weights (>5, >10, and >20) indicate good stability. The effective sample size (Σw)^2^/Σw^2^

**Supplementary Table S5. Entropy-balancing diagnostics: standardized mean differences before and after weighting**

| **Covariate** | **Abs SMD Before** | **Abs SMD After** |
| --- | --- | --- |
| Gestational age in weeks | 0.194 | 0.0 |
| Birth weight in grams | 0.080 | 0.0 |
| Sex | 0.223 | 0.0 |
| SGA | 0.236 | 0.0 |
| Twin | 0.274 | 0.0 |
| Pre-eclampsia | 0.278 | 0.0 |
| PPROM | 0.161 | 0.0 |
| Clinical chorioamnionitis | 0.224 | 0.0 |
| Nulliparity | 0.072 | 0.0 |
| Induced preterm delivery | 0.150 | 0.0 |

**Abbreviations**: Abs SMD, absolute standardized mean difference; PROM, preterm premature rupture of membranes. Entropy-balancing weights were generated separately for each exposure group to ensure that the weighted mean of each covariate in the control group was equal to the overall sample mean (moment matching). Absolute SMDs of 0.10 or less indicate acceptable balance; entropy balancing reduced every covariate to perfect mean balance (SMD = 0).

**Supplementary Table S6. IPTW-weighted sensitivity analysis: adjusted risk ratios for neonatal outcomes**

| **Outcome** | **No ANCS exposure Events n/Total (%)** | **ANCS-to-delivery interval ≤24 h Events n/Total (%)** | **ANCS-to-delivery interval >24 h–7 d Events n/Total (%)** | **ANCS-to-delivery interval ANCS ≤24 h aRR^a^ (95% CI)** | **ANCS-to-delivery interval ANCS >24 h–7 d aRR^a^ (95% CI)** |
| --- | --- | --- | --- | --- | --- |
| Mortality | 12/101 (11.9%) | 11/167 (6.6%) | 3/64 (4.7%) | 0.64 (0.29–1.44) | 0.34 (0.1–1.2) |
| Severe RDS | 34/101 (33.7%) | 55/167 (32.9%) | 23/64 (35.9%) | 0.87 (0.62–1.24) | 0.86 (0.55–1.34) |
| Severe NEC | 9/101 (8.9%) | 3/167 (1.8%) | 4/64 (6.2%) | 0.17 (0.05–0.62)**^b^** | 0.68 (0.21–2.22) |
| Severe IVH | 4/101 (4.0%) | 4/167 (2.4%) | 2/64 (3.1%) | 0.67 (0.17–2.65) | 0.84 (0.16–4.5) |
| Composite outcome | 43/101 (42.6%) | 61/167 (36.5%) | 24/64 (37.5%) | 0.82 (0.6–1.12) | 0.76 (0.5–1.15) |

**Abbreviation**s: ANCS, antenatal corticosteroids; aRR, adjusted risk ratio; CI, confidence interval; IPTW, inverse probability of treatment weighting; NEC, necrotizing enterocolitis; IVH, intraventricular hemorrhage; RDS, respiratory distress syndrome; composite outcome = mortality or severe NEC, IVH, or RDS; Reference group (No ANCS).
**^a^** Adjusted risk ratios (aRRs) were estimated using modified Poisson regression with a log link and HC3 robust variance, incorporating stabilized IPTW trimmed at the 1st and 99th percentiles and capped at 20. Treatment-only models (no additional covariates) were used per sensitivity specification. Each aRR compares the indicated ANCS-to-delivery interval with the no-ANCS reference group.

**^b^** CI excludes 1.

Effective sample sizes (ESS) by exposure group under IPTW were 85.9 (no ANCS), 161.3 (≤ 24 h), and 60.0.(> 24 h–7 d)

Outcomes were prespecified; no multiplicity adjustment was applied. Confidence intervals (95%) are reported without correction and should be interpreted accordingly. Weighted totals may appear non-integer due to weighting

**Supplementary Table S7. Exploratory economic evaluation assumptions**

| **Parameter** | **Value** | **Source** |
| --- | --- | --- |
| Antenatal Corticosteroids (ANCS) cost | Indian Rupees (INR) 50–100/course (~ US$0.58–$1.16) | Local hospital data |
| Necrotizing enterocolitis (NEC) treatment cost | INR 400,000/case (~ US$4,620) | Local hospital data, Narang et al. (2005) (1) |
| Number needed to treat (NNT) for NEC | 14 | Study results (7.1% risk reduction) |
| Savings per NEC case prevented | INR 398,600–399,300 (~US $4,604–$4,612) | Calculated (NEC cost minus ANCS cost for NNT=14) |
| Disability-adjusted life years (DALYs) averted per NEC case | 5–10 | Murray et al. (2015) (2), Singh et al. (2002) (3) Prinja et al. (2013) (4) |

Abbreviations: DALYs, disability-adjusted life years (DALYs); Indian Rupees (INR), number needed to treat (NNT).

Note: Costs were converted to US dollars using an exchange rate of 1 USD = 86.6 INR, based on mid-2025 averages. Costs reflect low- and middle-income setting (LMICs) constraints (e.g., shorter NICU stays and limited surgical access), lower than US estimates (approximately US$17,000 per case) (5). DALYs estimated assuming 25% NEC mortality (years of life lost, YLL, ~7.5 DALYs, 3% discount), 75% of survivors with disability weight of 0.2–0.3 for 10–20 years (years lived with disability, YLD, ~1–3) due to strictures, short bowel syndrome, and neurodevelopmental impairment (1,4). This post-hoc analysis is exploratory and carries uncertainty owing to limited long-term outcome data and potential cost variability.

# **References**

1. Narang A, Kiran PS, Kumar P. Cost of neonatal intensive care in a tertiary care center. Indian Pediatr (2005) 42:989-997.
2. GBD 2013 DALYs and HALE Collaborators, Murray CJ, Barber RM, et al. Global, regional, and national disability-adjusted life years (DALYs) for 306 diseases and injuries and healthy life expectancy (HALE) for 188 countries, 1990-2013: quantifying the epidemiological transition. Lancet. 2015;386(10009):2145-2191. doi:10.1016/S0140-6736(15)61340-X
3. Singh J, Sinha S. Necrotizing enterocolitis--an unconquered disease. Indian Pediatr (2002) 39:229-237.
4. Prinja S, Manchanda N, Mohan P. Cost of neonatal intensive care delivered through district level public hospitals in India. Indian Pediatr (2013) 50:839-46. doi: 10.1007/s13312-013-0234-y.
5. WHO ACTION Trial Collaborators. Antenatal dexamethasone for improving preterm newborn outcomes in low-resource countries: a cost-effectiveness analysis of the WHO ACTION-I trial. Lancet Glob Health (2022) 10:e1523-33. doi: 10.1016/S2214-109X(22)00340-0
